# Aesthetic Outcomes and Peri-Implant Health of Angled Screw Retained Implant Restorations Compared with Cement Retained Crowns: Medium Term Follow-Up

**DOI:** 10.3390/jfb12020035

**Published:** 2021-05-16

**Authors:** Livia Nastri, Ludovica Nucci, Vincenzo Grassia, Rino Miraldi

**Affiliations:** 1 Multidisciplinary Department of Medical-Surgical and Dental Specialties, University of Campania “Luigi Vanvitelli”, 80138 Naples, Italy; ludovica.nucci@unicampania.it (L.N.); vincenzo.grassia@unicampania.it (V.G.); 2 Private Practice, 80122 Naples, Italy; rinomiraldi@gmail.com

**Keywords:** implant restorations, aesthetic outcome, angled screw channel, cemented implant crowns, white esthetic score, pink esthetic score

## Abstract

Single tooth implant restorations in the aesthetic area are a demanding challenge. If a complete osseointegration is mandatory, the final result has to result in a higher standard of biomimetic and soft tissue health among natural teeth. This outcome is traditionally pursued by cementing crowns over individualized abutments. However, in recent years, the need for controlling peri-implant health and the preference towards a retrievable solution has led to an increase in screw-retained crowns, which is not always applicable when the implant axis is not ideal. In the aesthetic area, the use of a novel technical solution represented by the angled screw channel (ASC) of the abutment has been proposed in order to match the advantages of the screwed solution with the aesthetic demands. The aim of this study was to compare ASC crowns to cemented crowns (CC) in single implant restorations using the white esthetic score (WES) and pink esthetic score (PES) at the crown delivery and at a follow-up of a minimum of 2 years. Peri-implant health and marginal bone loss (MBL) were also evaluated. The mean follow-up was 44.3 months, with a mean MBL of 0.22 mm in the ASC group and 0.29 mm in the CC group. The total WES/PES score was 16.6 for ASC, compared with 17.3 for CC at baseline, and 16.2 and 17.1, respectively, at follow-up. Both of the groups reached a high WES/PES, and this was maintained over time, without signs of peri-implant diseases or bone loss, regardless of the choice of connection. In conclusion, ASC can be adopted in cases where the implant axis is not ideal, with aesthetic and functional results that are comparable to implants restored by cemented crowns.

## 1. Introduction

In the last few years, the debate about the choice between screw- and cement-retained implant prostheses has been ongoing, without definitive answers. The main advantage of screw-retained restorations is the predictable retrievability that can be achieved without damaging the restoration or the fixture, so that screw retention becomes more necessary in extensive cases where prosthesis may need more maintenance [1,2]. Moreover, in cases where biologic complications are anticipated (i.e., in periodontal patients), screw-retained restorations are preferred to allow an easy removal of the restorations, both for diagnostic purposes and for treatment [1].

Although when the implant is placed in the ideal position, predictable aesthetics can be achieved with either screw- or cement-retained restorations, screw-retained implant-supported restorations require precise placement of the implant in order to achieve optimal results, while the use of cemented restorations allows for greater freedom in implant placement [3]. Nevertheless, some authors have reported a higher incidence of gingival inflammation when using cement-retained prosthesis, especially when the restoration margin is deeper than 3 mm subgingivally, because of the difficulties in cement remnants removal [4]. This depth is particularly common in the anterior region when it is recommended to place the implant 3 to 4 mm apical to the cementoenamel junction or the facial gingival margin of adjacent teeth to develop a proper emergence profile. For this reason, and for the need for retrievable crowns in case of peri-implant diseases, screw-retained crowns are claimed to be preferable. However, in many cases, in the aesthetic region even in a well-designed implant positioning, may lead to a screw channel projection on the vestibular side or on the margin of the future crown [5,6].

**Figure 1:**
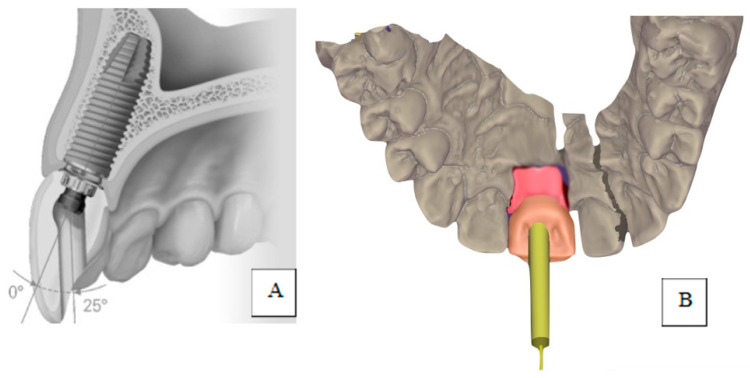
Schematic drawing and digital cast of the angled screw channel abutment. (**A**) The design of the angled screw channel (ASC) abutment allows the screw access hole to be angulated on the lingual side of the restoration. Effective torque can be applied at any angle from 0 to 25 degrees with regard to the implant axis. (**B**) The access hole can be moved to a position that is close to the palatal cingulum.

In the few last years, a novel technical component (angled screw channel (ASC)) has been used that could potentially help where screw retention is preferable, but the axis of the abutment is not ideal for screw retention. These kinds of abutments are connected by a screw that can be screwed off-axis, and allow for fixing abutments from a palatal hole [7] (Figure 1).

To the best of our knowledge, while different studies have investigated the mechanical behaviour of angulated access screws [8,9,10], few studies have compared the aesthetic and health of implant restorations fixed using angled screw channel retention or cemented in the aesthetic area.

The aim of this retrospective study is to compare the aesthetic results and peri-implant health in these two groups of single implant restorations.

## 2. Materials and Methods

**Scheme 1:**
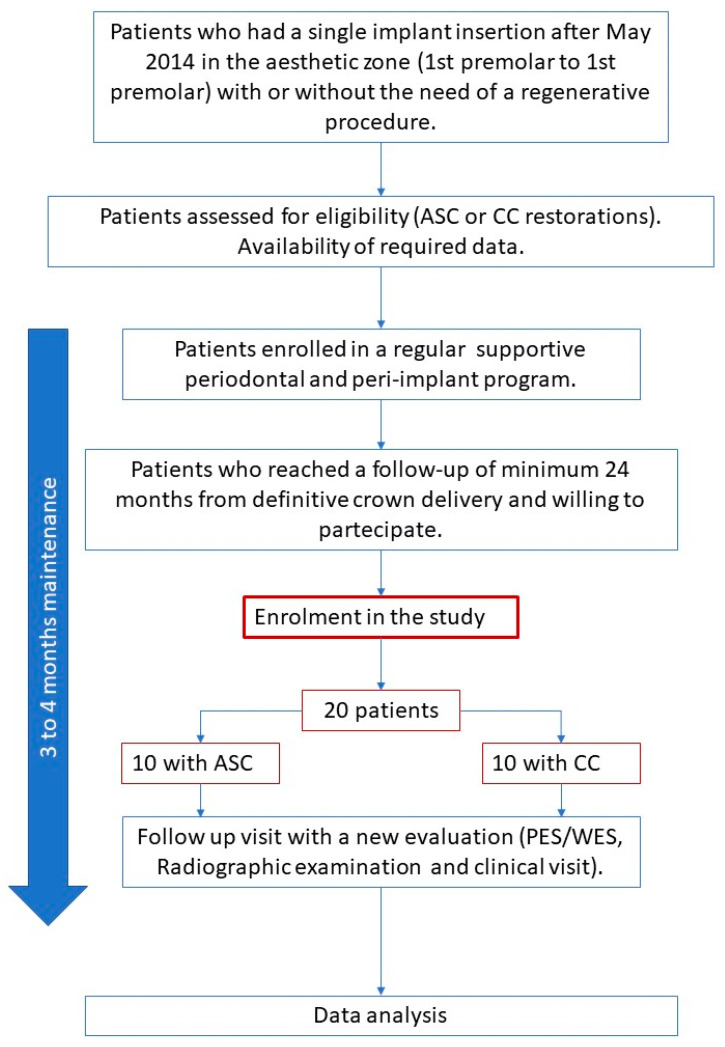
Schematic outline of patients recruitment and follow-up.

A sample of patients was selected from the records of implant patients based on the following criteria: (1) restoration with a single implant in the aesthetic area (defined as the area from the first premolar to the first contralateral premolar of the upper and lower jaw), (2) treatment that occurred after May 2014 (when ASC was firstly available), (3) the implant axis was not suitable for a straight screw-retained crown (implants were restored using a cemented restauration on a personalized abutment (CC) or angled screw channel retained crown (ASC) (Nobel Biocare Zürich, Switzerland), (4) a minimum of 24 months passed between the crown delivery and the follow-up visit, (5) there was high quality intraoral photography available from the time of the crown delivery (where the corresponding contralateral tooth was visible, the interproximal papillas were appreciable, and the marginal soft tissues were visible), (6) periapical radiography of the implant at the definitive crown delivery was available, and (7) clinical records of peri-implant probing depth and bleeding on probing were available (Scheme 1).

All of the implant patients were enrolled in a periodontal/peri-implant maintenance program with 3 to 4 months intervals. Complications or non-planned interventions were recorded during maintenance.

**Figure 2:**
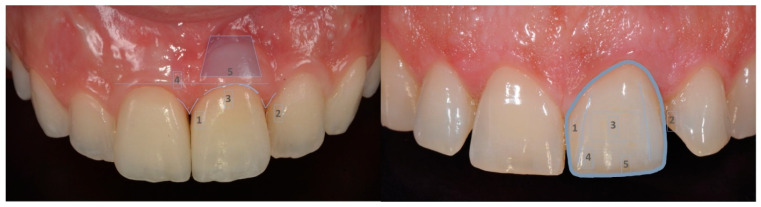
Pink esthetic score (PES) and white esthetic score (WES) according to Belser et al. [11]. For each site/parameter listed below and graphically indicated in the pictures, a score of 0, 1, or 2 was accorded to the restoration and its peri-implant surrounding soft tissue. PES sites: (1) mesial papilla, (2) distal papilla; (3) curvature of the facial mucosa; (4) level of facial mucosa; (5) root convexity/soft tissue colour and texture. WES parameters: (1) tooth form; (2) outline/volume; (3) colour (hue/value); (4) surface texture; (5) translucency/characterization. A score of 2 corresponds to “no discrepancy”, a score of 1 corresponds to “minor discrepancy”, and a score of 0 corresponds to “major discrepancy”, referring to the contralateral natural tooth. A maximum score of 10 for PES and a maximum score of 10 for WES may be accorded in an ideal situation.

Peri-implant tissues and restorations of the recruited patients were evaluated using the Pink Esthetic Score (PES) and White Esthetic Score (WES), according to Belser et al. [11], based on the intraoral pictures taken at the definitive crown delivery (Figure 2).

**Figure 3:**
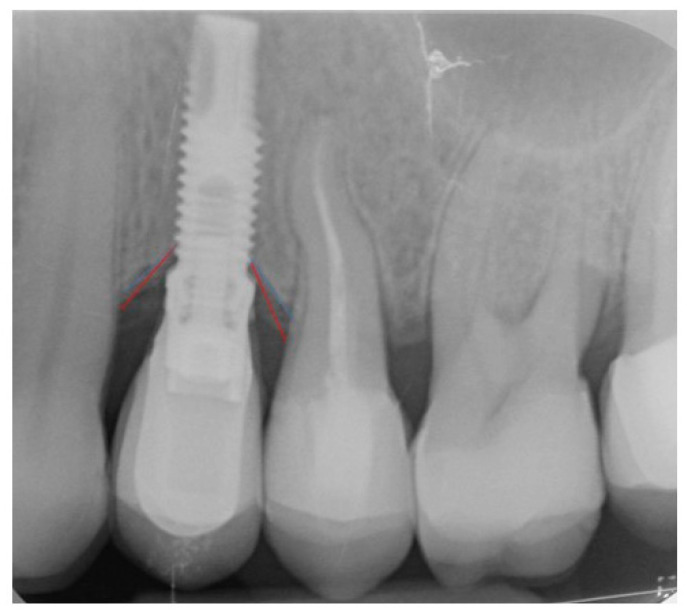
An example of the superimposing of a periapical radiograph to evaluate marginal bone loss.

After a minimum of 24 months of follow-up, the probing depth and bleeding on probing were measured, a periapical X-ray was taken, and the PES and WES were calculated on the current intraoral pictures. Marginal bone loss (MBL) was calculated on the intraoral radiographs when the follow-up X-rays were superimposed on the baseline X-ray and were measured using a diagnostic software (Planmeca Romexis, Helsinki, Finland). Measurements were taken after calibration on the implant length, highlighting the bone crest profile (Figure 3). All of the subjects gave informed consent for inclusion before they participated in the study, and during the course of the research. The study was conducted in accordance with the Declaration of Helsinki.

Enrolled patients were divided into two groups: the CC group for patients with implants that were restored with cemented crowns on personalized abutments, and the ASC group for patients with implants restored with angled screw channel abutments and crowns. Data were analysed using Student’s *t*-test for continuous variables and chi-squared test for categorical variables, and were considered significant if *p* < 0.05.

## 3. Results

Twenty patients who had a single implant inserted from September 2014 to July 2016 in the aesthetic area, requiring a correction of the axis, met the inclusion criteria; 12 were female and 8 were male, with a mean age of 47.5 years old (25–67 years old). Four implants were placed in the position of the central incisor, eight implants in an upper lateral position, four in an upper canine position, one in an upper first premolar position, and three in a lower lateral position. None of the studied implants failed during the follow-up period, which was a mean time of 44.3 (±11.5) months. Patients were divided into two groups according to their mean of prosthetic fixation of either CC or ASC. Ten patients received CC and 10 ASC. The mean follow-up was 36.4 (±10.3) months for the ASC group and 52.3 (±5.7) months for the CC group, with a statistically significant difference. Among the recruited patients, six out of ten restorations in the ASC group were performed after a bone regeneration, compared with three in the CC group. Although noteworthy, this difference did not reach statistical significance (Table 1).

WES at baseline was 8.7 (±1.5) for ASC and 8.3 (±0.9) for CC, while PES was 7.9 (±0.73) and 9 (±0.66), respectively. PES was statistically significantly different in ASC and CC at baseline. At follow-up, WES was 8.5 (±1.35) for ASC and 8.3 (±0.94) for CC, while PES was 7.7 (±0.82) and 8.8 (±0.63), respectively. The difference in PES values between the two groups reached statistical significance (Table 1).

Bleeding on probing was present in two implant sites at baseline on cemented crowns and in four implant sites at follow-up (two CC and two ASC), without any statistical significance (*p* = 0.446). The peri-implant probing depth remained stable in both groups. At baseline, PD was 3.7 mm (±0.48) in the ASC group and 3.3 mm (±0.48) in the CC group (*p* = 0.08), while a PD of 4 mm (±0.66) in the ASC group and a PD of 3.7 mm (±0.82) were recorded at follow-up (*p* = 0.383; Table 2).

The mean MBL was −0.25 (±0.15) mm at the end of the follow-up, with a value of −0.22 (±0.19) mm in the ASC group and a value of −0.29 (±0.11) mm in the CC group (Table 2).

No technical complications occurred during follow-up, none of the crowns were decemented or unscrewed, and there was no need for unplanned technical interventions. Only two minor biological complications (one in the CC group and one in the ASC group) were recorded as mucositis, and were treated and solved during routine maintenance.

## 4. Discussion

All of the restorations reached a more than satisfying WES/PES score both at baseline and at follow-up. The cemented crowns had a better PES score than the angled screw channel retained crowns (8.8 vs. 7.7 at follow-up). However, the screw retained crowns were chosen for implants inserted in the augmented site in 60% of the cases, compared with only 30% of the cemented crowns. This could justify, in part, the different outcomes of the PES values. PES of ASC were also evaluated by Shi et al. [12] in a recent study on immediate post-extractive implants inserted with a flapless procedures. At the baseline examination, PES in their study was 8.35 ± 1.53 in the ASC group and 8.30 ± 1.38 in the CC group, while at the one-year observation, the PES value was 8.96 ± 0.88 in the ASC group and 8.98 ± 0.62 in the CC group. Even though this is slightly higher than the PES value in our study, it has to be considered that ideal conditions are a prerequisite for an immediate post-extractive insertion with a flapless procedure. Our cases were not ideal at the beginning of the treatment; most of them were periodontal patients requiring bone regeneration to obtain a satisfying quantity of bone in order to insert the implant. WES/PES was also used in a very recent study to evaluate 1 year and 5 years follow-up in patients enrolled in a randomized clinical trial comparing the immediate loading and delayed loading of single-tooth implants. The authors showed a progressive increase in aesthetics with time, related to the gradual papillae formation and the healing process of the mucosa [13]. However, according to the study design, none of the teeth replaced by implants were lost for periodontal reasons, while in our study, most of the patients lost their teeth because of periodontitis, and this aspect may have influenced not only the surgical phases, but also the expectations on tissue stability/improvements over time.

In our study, the mean follow-up was longer for CC, because of the increasing tendency towards using screw retained restorations in recent years compared with the tendency towards cementing the crowns in the first period of the study. It needs to be reinforced that this study reported on implants that could not be restored with in-axis screw retention. This condition may happen despite a bone augmentation procedure and/or correct surgical implant insertion, and can be corrected by a cemented crown on a customized abutment or by an angled screw retained customized abutment [14].

No technical complications occurred in either of the groups and no unplanned interventions took place, in contrast with Wittneben et al. [15] who reported that screw-retained reconstructions exhibited fewer technical and biologic complications. The crowns in the ASC group did not show additional technical problems related to unwanted screw loosening, in contrast with the study by Opler et al. [8], who revealed low reverse torque values for angled screw abutments after tightening, at least at high angulations (>25°), as well as with Hu et al. [9] in another laboratory study on reverse torque value. In our study, the screw angulation was not measured, but all of the screws were tightened according to the manufacturer instructions (up to a torque of 35 N cm), without any screw loosening in the follow-up of 52 months in the ASC group. This was in agreement with Pol et al. [16], who analysed the outcomes of posterior implants restored by ASC, and showed no complications at the one year follow-up such as a loosening of the restoration. In a previous retrospective study on ASC, only two restorations out of 84 showed complications, one was a loose screw and the other a ceramic fracture [17]. In another laboratory study, ASC performed similar to conventional straight-line screw access with respect to the torque value [10]; this could indicate some differences among the different methodologies and materials employed to perform the studies.

The presence of screw retention could be useful in cases of peri-implant diseases or technical complications that require the removal of the crown, like chippings. However, during the follow-up of this study (up to 58 months), the crowns did not need to be unscrewed or decemented in order to perform maintenance.

The other clinical and radiographic parameters did not show any significant differences between the studied groups. The mean marginal bone loss was low in the two groups and within the ranges reported by other authors for similar follow-up times [18,19]. The results observed in the studied groups indicate that MBL was not affected by the angulation of the screw channel, in accordance with Anitua et al. [20] in a recent publication and in agreement with Shi et al., even though they used different surgical and prosthetic procedures [12]. The marginal bone loss was lower than that reported by Vigolo et al. [21], who found slightly worse behaviour of straight screw retained crowns compared with cemented crowns in single implant restorations, although in their study, the authors restored mainly posterior missing teeth.

No differences were appreciated for the probing depth and bleeding on probing between the two groups. The presence of bleeding at definitive crown delivery was noticed in two cases in the cemented group, which could be related to cementation procedures and cement removal, and were without any subsequent complications or statistical significance. Overall health maintenance in the medium follow-up was found to be adequate in order to foresee long-term good peri-implant health stability.

Angled screw channel retention was shown to be a suitable alternative to customized abutments and cemented restorations for when the implant axis is not ideal. Especially with the use of bone-level implants, the cementation procedure could require significant attention and should be carried out with great caution in order to avoid cement remnants in the mucosa [2]. ASC may be chosen in the cases when an angle correction of up to 25 degrees is required [22], and may offer some advantages in cases of technical and biological complications.

As the aesthetic outcome of the ASC restorations in our study was as satisfying as in the cemented crowns group, the ASC abutment could successfully be used where the residual bone after tooth loss does not allow for implant insertions with planned prosthetic crowns that are perfectly in axis, and where the screw emergence would lay in a vestibular or a marginal site.

One of the main limitations of this study is its retrospective nature, which did not allow for selecting cases according to the same characteristics. The choice of the retention modality was made according to the clinical, radiographic, and prosthetic conditions.

Another limitation is the use of WES/PES evaluation at follow-up, when, in some of the cases, the restoration became different from the natural contralateral tooth because of ageing and the appearance changes of the latter. Even if PES/WES is still the most widely accepted tool to assess the aesthetic outcomes of single implant-supported crowns in the maxillary anterior region from a clinician perspective [23], the score is probably more suitable for ideal cases in younger patients. The cases included in this study were not always ideal in terms of natural teeth characteristics and position, and had previous bone loss, often requiring bone augmentation procedures before inserting the implants. Notably, this study is limited by its small sample size, which may have resulted in some bias, and, as a consequence, small differences between groups may not have been detected, so larger, more well-designed studies are needed. However, the study was intended to identify the different aesthetic outcomes and health status changes of implants with different crown retention means. No significant differences were noticed with this investigation, suggesting that angled screw channel abutment can be equally suitable for a satisfying aesthetic and functional results as customized abutments for cemented crowns.

## Figures and Tables

**Table 1:** Sample characteristics and aesthetic findings in the angled screw channel crowns group and the cemented crowns group. WES—white esthetic score; PES—pink esthetic score; ±SD—standard deviation. Data are analysed using Student’s *t*-test. ^§^ indicates that a chi squared test is only applied for “regeneration needed”. Statistical significance is set at *p* < 0.05.

Type of Retention	Mean Patient Age (Years)	Regeneration Needed	Mean Follow-Up (Months; ±SD)	Mean WES (Baseline; ±SD)	Mean PES (Baseline; ±SD)	Mean WES (Follow-Up; ±SD)	Mean PES (Follow-Up; ±SD)
Angled Screw Channel crowns	42.8	6/10	36.4 (±10.3)	8.7 (±1.5)	7.9 (±0.73)	8.5 (±1.35)	7.7 (±0.82)
Cemented Crowns	51.9	3/10	52.3 (±5.7)	8.3 (±0.9)	9(±0.66)	8.3 (±0.94)	8.8 (±0.63)
*p* = (* < 0.05)	1.7	0.369 ^§^	* < 0.0005	0.48	* 0.026	0.7	* 0.036

*: statistically significant.

**Table 2:** Peri-implant probing depth, bleeding on probing and marginal bone loss in the Angled Screw channel crowns group and the cemented crowns group. PD—probing depth; BOP—bleeding on probing; MBL—marginal bone loss; ±SD—standard deviation. Data are analysed using Student’s *t*-test. ^§^ indicates a chi squared test is applied only for “BOP”. Statistical significance is set at *p* < 0.05.

Type of Retention	Mean PD Baseline (mm; ±SD)	*p* Follow Up (mm; ±SD)	BOP Baseline (+/Total)	BOP Follow Up (+/Total)	MBL (Follow Up vs. Baseline, mm; ±SD)
Angled Screw Channel Crowns	3.7 (±0.48)	4 (±0.66)	0/10	2/10	−0.25 (±0.15)
Cemented Crowns	3.3 (±0.48)	3.7 (±0.82)	2/10	2/10	−0.29 (±0.11)
*p* = (* < 0.05)	0.08	0.383	0.446 ^§^		0.32

*: statistically significant.

## Data Availability

The data presented in this study are available on request from the corresponding author.
